# Giant occipital encephalocele complicated with obstructive hydrocephalus: A case report

**DOI:** 10.1016/j.amsu.2022.104176

**Published:** 2022-07-12

**Authors:** Durga Neupane, Alok Dahal, Nimesh Lageju, Lokesh Shekher Jaiswal, Prabin Bhusal, Akash Gurung, Krishnaraj Aryal, Sagar Panthi

**Affiliations:** a Department of Surgery, B.P. Koirala Institute of Health Sciences, Dharan, Nepal; b Department of Surgery (Division of Neurosurgery), B.P. Koirala Institute of Health Sciences, Dharan, Nepal; c Department of Surgery (Division of CTVS), B.P. Koirala Institute of Health Sciences, Dharan, Nepal; d Maharajgunj Medical Campus, Institute of Medicine, Kathmandu, Nepal

**Keywords:** Case report, Encephalocele, Neural tube defect, Resection, Hydrocephalus, Complication

## Abstract

**Introduction:**

and importance: An encephalocele is a type of congenital neural tube defect defined by herniation of intracranial contents via a cranial defect. When an encephalocele is greater than the size of the head, it is referred to as a "giant encephalocele." The occurrence of encephalocele has been documented to be 1–4 instances per 10,000 live births. Surgery is challenging.

**Case presentation:**

A 1-month-old baby boy sustained a huge swelling on the back of his head since birth, and it increased gradually over time. On examination, he had a huge occipital swelling measuring about 20 × 15 × 17 cm in size. A diagnosis of giant occipital encephalocele was established. Surgical excision and repair was done. After 1 month, he developed obstructive hydrocephalus and a ventriculo-periotoneal shunting was performed. On regular follow-up, he is in a good state of health.

**Discussion:**

Surgery imposes challenges for the anaesthesiologists and neurosurgeons due to its complex site, enormous size, intraoperative blood loss, and prolonged anaesthesia. A team approach is necessary for its successful treatment.

**Conclusions:**

Based on our experience, we would like to deliver following recommendations in the surgical management of giant encephalocele. **Surgery should be done quickly to ensure good prognosis.** Proper positioning, efficient intubation, infections and sepsis control should be emphasized. Also, fortification of food with folic acid, as well as increased education and awareness of women on the need for antenatal care may also decrease the risk of this disease.

## Highlights

• An encephalocele is a type of congenital neural tube defect.
• Surgery imposes challenges for the anaesthesiologists and neurosurgeons.
• A team approach is necessary for its successful treatment.
• Proper positioning, efficient intubation, infections and sepsis control should be emphasized.
• Fortification of food with folic acid may also decrease the risk of this disease.

## 1 Introduction

An encephalocele is a type of congenital neural tube defect (NTD) defined by herniation/protrusion of intracranial contents (meninges, brain, and portion of the ventricles) via a cranial defect [1]. Encephalocele is formed by an inability of the cranial section of the developing neural tube to close around the first few weeks of fetal life. When an encephalocele is greater than the size of the head, it is referred to as a "giant encephalocele." [2] The occurrence of encephaloceles has been documented to be 1–4 instances per 10,000 live births, with the occipital region being the most prevalent location (75% of cases). Frontoethmoidal (13–15%), parietal (10–12%), and sphenoidal are the following sites [3]. Encephaloceles are linked with significant morbidity and death; despite preoperative and postoperative treatments, occipital encephaloceles have the poorest prognosis [4]. Encephaloceles are classified as occipital, anterior, parietal, temporal, or vertex encephaloceles based on their morphological region. Encephaloceles are also classified on the anatomical position of the gap in the cranium [5]. Early excision and repair are advised because it drastically minimises well-known problems such as cerebrospinal fluid (CSF) leak and diminished intelligence level [6].

Accompanying hydrocephalus is relatively common with giant occipital encephaloceles or develops after surgical intervention, and CSF diversion in the form of a ventriculoperitoneal shunt is also needed [7,8].

We report a case of giant encephalocele in a 1-month-old baby boy. Surgical excision and repair was done. After 1 month, he developed obstructive hydrocephalus and a ventriculo-periotoneal (VP) shunting was performed. On regular follow-up, he is in a good state of health. This case report is reported according to SCARE guideline [9].

## 2 Case report

**Fig. 1:**
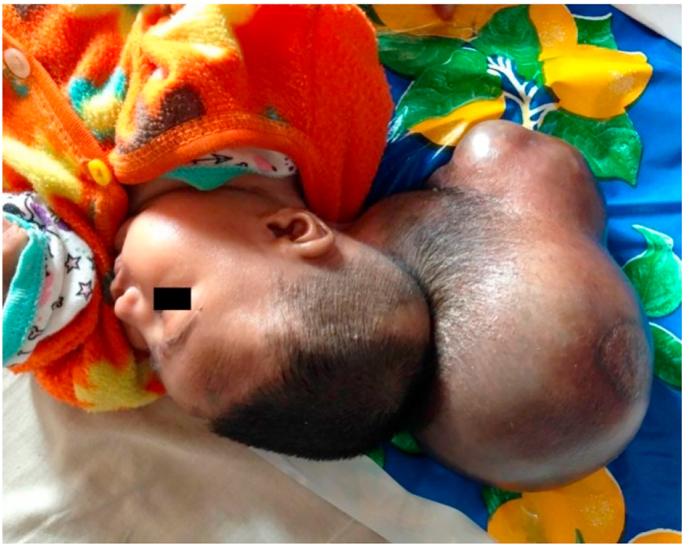
A giant occipital encephalocele measuring 20 × 15 × 17 cm.

**Fig. 2:**
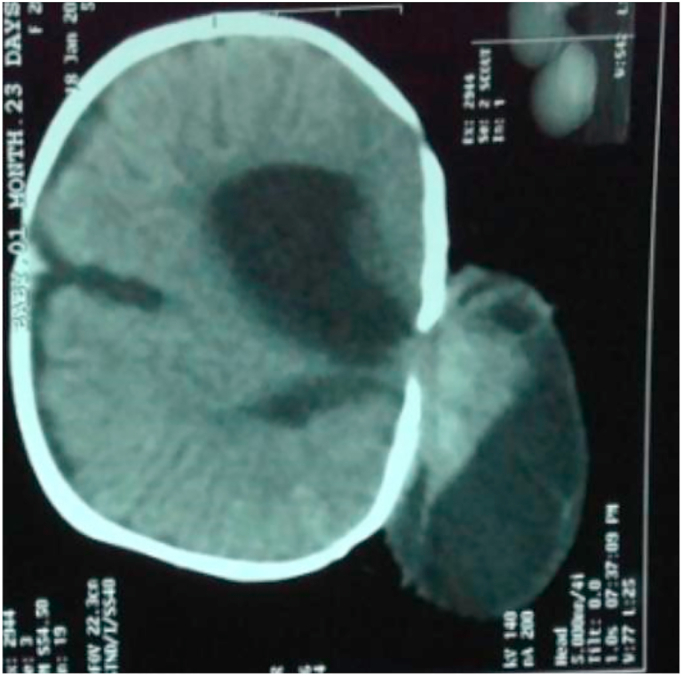
NCCT head showing a cerebrospinal fluid (CSF) containing sac with the brain parenchymal tissue herniating through a defect in posterior occipital bones in midline.

A 1-month-old baby boy sustained a huge swelling on the back of his head since birth, and it increased gradually over time. Baby was delivered via caesarean section at term. On examination, he had a huge occipital swelling measuring about 20 × 15 × 17 cm in size (Fig. 1). Swelling was cystic and transilluminated. The overlying skin was intact. Neurological examination and baseline investigations were normal. **Family history of any neural tube defects was negative. No any associated anomalies were reported. Genetic history was unremarkable. Environmental exposure and toxins were unremarkable. Antenatal history was normal and there was no evidence of encephalocele by antenatal ultrasound. His mother had a regular intake of folic acids antenatally.** His NCCT head reported a cerebrospinal fluid (CSF) containing sac with the brain parenchymal tissue herniating through a defect in posterior occipital bones in midline (Fig. 2). A diagnosis of giant occipital encephalocele was established. Surgical excision and repair was done**. Pre-operative counselling was done. Various challenges like bleeding, tonsillar herniation and difficult intubation were kept in mind.** After 1 month, he developed obstructive hydrocephalus and a VP shunting was performed. On regular follow-up, he is in a good state of health**. He has not reported any infections and the shunt is advised to be intact lifelong.**

## 3 Discussion

The understanding of the etiopathogenesis of NTD and its association with folic acid deficiency has made its supplementation a vital step in the control of this condition. Supplementation of folic acid, routine prenatal care and legalization of abortion in developed countries has led to a diminish in encephalocele and other neural tube defects. In developing countries, fortification of food items with folic acid is not done routinely. This is why, a higher incidence of encephalocele and giant encephalocele exists in developing countries [10]. However, according to her antenatal history, folic acid supplementation to her mother was done according to national protocol.

The exact cause of encephalocele is unknown, and it is probably multifactorial, including both genetic and environmental factors. Individuals with a family history of neural tube defects (spina bifida, anencephaly) are more likely to develop encephalocele. Certain researchers have emphasized that non-specific environmental factors (such as hyperthermia, aflatoxins, genetic background, mother's lack of nutrition or other environmental factors) are involved in the development of encephalocele [11]. Even certain toxins or infections might play a role in its genesis. However, no any genetic, environmental factors and family history was supportive of the diagnosis in our case.

Intraoperatively intubation of such patients during the surgery can be a major challenge. [12,13]  While some patients are positioned in the lateral position, others prefer supine position with the use of a head roll and large shoulder support to enable neck extension. Several challenges of patient care include nursing care, risk of rupture and meningitis prior to the surgery. At times, brain tissue can also herniate and the skin may even endure pressure necrosis [10]. Because of the large size of encephalocele in our case, lateral intubation was done.

Considering the fluid in the encephalocele sac as a third space, rapid patient infusion with warm fluids should be made when the sac is opened, and fluid volume lost. Infiltrating the wound with lidocaine and adrenaline improves haemostasis. The operation site can also be injected with copious amounts of water (the hydro-dissection technique) in addition to the lidocaine with adrenaline. An elliptical incision is made and careful haemostasis is secured with monopolar and bipolar diathermy. Transfusion should be available as required. However, very few cases need blood products [10]. Adequate fluids and appropriate incision was done in our case. Blood transfusion was not required.

Although the patients are mostly placed in a prone position, the head is usually turned to one side because of the size of giant encephalocele. Routine cleaning and draping of the patient is necessary. The cleaning can be technically challenging sometimes due to its large size. The scalp incision should be high enough. It is made with the scalpel and diathermy after infiltration with either adrenaline in saline or diluted lidocaine with adrenaline. Haemostasis is then maintained [10].

Intraoperative and post-operative temperature control is key to avoid complications and ensure successful early post-operative outcome. Hypothermia was the main cause of mortality in one of the largest series of giant encephalocele [7]. The review of the literature has shown that it may be associated with Dandy Walker malformation, cleft lip and palate, microcephaly and micrognathia [[13], [14], [15]]. Temperature was strictly regulated in our case. No any associated anomalies were noted.

Depending on the size of the defect, several methods of closure of giant encephalocele have been described in the literature. Small defects are managed with simple excision of the encephalocele with closure of the dura and pericranium, followed by closure of the subcutaneous tissue and skin. In large defects, various methods have been practised ranging from simple closure to autologous graft, tantalum and latisimus dorsi free flap [[16], [17], [18]]. Simple closure was adopted in our case.

A review concludes that babies who undergo operative intervention in the first month of life are likely to have poorer early post-operative outcomes due to hypothermia. Early surgery is sometimes favoured because of the psychosocial impact on the family. However, since most of these cases are seen in developing countries where paediatric, anaesthetic and neonatal intensive care may not be optimal, they have recommended a relatively later timing of surgery [10].

Surgical intervention is the best option for treating occipital encephalocele, and the most optimal time for surgery is between birth and 4 months. If the skin layer covers and protects the lesion, the intervention may be delayed within a few months; otherwise, without skin protection, the surgery should be done as quickly as possible [19].

Some of the challenges to us were the risk of bleeding, development of hydrocephalus and tonsillar herniation. The overlying skin was intact in our case and the patient presented to us at 1 month. Therefore, we planned the surgery at 1 month irrespective of neonate's weight. Had he been diagnosed early, we would have operated quickly as soon as possible.

Amongst new-borns with occipital encephalocele, 60-70% of the patients are at risk of developing hydrocephalus, which should be treated by application of the ventriculoperitoneal (VP) shunt [20]. In the majority of cases, hydrocephalus develops postoperatively. One of the mechanisms causing hydrocephalus may be torsion of the aqueduct of Sylvius or aqueductal stenosis. Surgical repair of the occipital encephalocele sac may provoke hydrocephalus due to changes in the CSF dynamics [21]. Hydrocephalus and microcephaly are poor prognostic factors associated with developmental delays and should be treated with expansible cranioplasty and VP shunt [20]. In line with the aforementioned finding, he developed obstructive hydrocephalus post-operatively and VP shunting was done. We had advised the patient's party regarding this pre-operatively and planned to keep VP shunt intact lifelong. If evidence of infections occur in the future, then it will possibly be removed.

Some limitations to our study include the inability to diagnose it prenatally and inability to prevent develop obstructive hydrocephalus.

## 4 Conclusions

Based on our experience, following recommendations in the surgical management of giant encephalocele can be inferred. **Surgery should be done quickly within few days of presentation to ensure good prognosis.** Temperature control should be maintained. Intravenous fluid resuscitation is an absolute necessity if sac ruptures. Proper positioning, efficient intubation, infections and sepsis control should be emphasized. Surgery imposes challenges for the anaesthesiologists and neurosurgeons due to its complex site, enormous size, intraoperative blood loss, and prolonged anaesthesia. **Pre and post-operative complications regarding development of hydrocephalus should be counselled. Antenatal diagnosis should be encouraged.** Also, fortification of food with folic acid, as well as increased education and awareness of women on the need for antenatal care may also decrease the risk of this disease. Definitive steps should be taken to legislate laws that will enforce fortification of food with folic acid especially in the developing countries like Nepal.

## Consent for publication

Written informed consent was obtained from the patient's father for publication of this case report and accompanying images as the case was minor. A copy of the written consent is available for review by the Editor-in-Chief of this journal on request.

## Ethical approval

Not required.

## Sources of funding

None.

## Author contribution

Durga Neupane(DN), Alok Dahal(AD), Lokesh Shekher Jaiswal(LSJ) = Study concept, Data collection, and surgical therapy for the patient. Durga Neupane(DN), Alok Dahal(AD), Nimesh Lageju(NL), Krishnaraj Aryal(KA) = Writing- original draft preparation. Durga Neupane(DN), Alok Dahal(AD), Prabin Bhusal(PB), Sagar Panthi(SP) = Editing and writing. Durga Neupane(DN), Alok Dahal(AD), Akash Gurung(AK) = senior author and manuscript reviewer. All the authors read and approved the final manuscript.

## Trail registry number

1. Name of the registry:

2. Unique Identifying number or registration ID:

3. Hyperlink to your specific registration (must be publicly accessible and will be checked):

## Guarantor

Durga Neupane.

## Annals of medicine and surgery

The following information is required for submission. Please note that failure to respond to these questions/statements will mean your submission will be returned. If you have nothing to declare in any of these categories then this should be stated.

## Consent

Written informed consent was obtained from the patient's father for publication of this case report and accompanying images as the case was minor. A copy of the written consent is available for review by the Editor-in-Chief of this journal on request.

## Provenance and peer review

Not commissioned, externally peer-reviewed.

## Declaration of competing interest

None.
